# On the buckling of an elastic holey column

**DOI:** 10.1098/rspa.2017.0477

**Published:** 2017-11-15

**Authors:** C. G. Johnson, U. Jain, A. L. Hazel, D. Pihler-Puzović, T. Mullin

**Affiliations:** 1 Manchester Centre for Nonlinear Dynamics and School of Mathematics, University of Manchester, Oxford Road, Manchester M13 9PL, UK; 2 Manchester Centre for Nonlinear Dynamics and School of Physics and Astronomy, University of Manchester, Oxford Road, Manchester M13 9PL, UK; 3 Physics of Fluids Group, University of Twente, 7500 AE Enschede, The Netherlands; 4 Mathematical Institute, University of Oxford, Woodstock Road, Oxford OX2 6GG, UK

**Keywords:** periodic structures, bifurcations, mechanical metamaterials

## Abstract

We report the results of a numerical and theoretical study of buckling in elastic columns containing a line of holes. Buckling is a common failure mode of elastic columns under compression, found over scales ranging from metres in buildings and aircraft to tens of nanometers in DNA. This failure usually occurs through lateral buckling, described for slender columns by Euler’s theory. When the column is perforated with a regular line of holes, a new buckling mode arises, in which adjacent holes collapse in orthogonal directions. In this paper, we firstly elucidate how this alternate hole buckling mode coexists and interacts with classical Euler buckling modes, using finite-element numerical calculations with bifurcation tracking. We show how the preferred buckling mode is selected by the geometry, and discuss the roles of localized (hole-scale) and global (column-scale) buckling. Secondly, we develop a novel predictive model for the buckling of columns perforated with large holes. This model is derived without arbitrary fitting parameters, and quantitatively predicts the critical strain for buckling. We extend the model to sheets perforated with a regular array of circular holes and use it to provide quantitative predictions of their buckling.

## 1. Introduction

**Figure 1.**
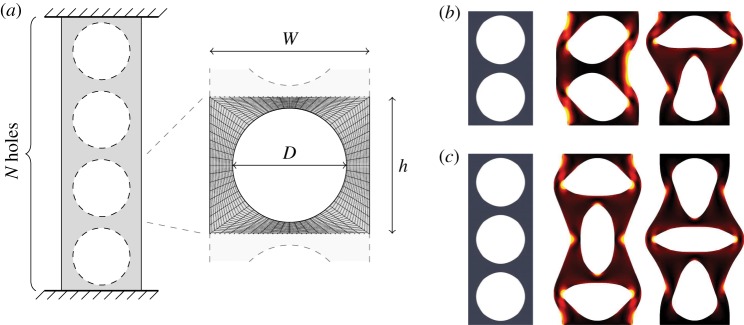
(*a*) Sketch of the undeformed configuration of a holey column, indicating the geometry of the ‘unit cells’ and a typical finite-element mesh. (*b*) Illustration of the Euler mode (centre) and alternating mode (right) of a deformed two-hole column. Each buckled state occurs through a pitchfork bifurcation, which breaks a reflection symmetry of the uncompressed column. (*c*) The alternating mode with an odd number of holes, which does not break a reflection symmetry. In (*b*),(*c*), the shading indicates the distribution of strain energy in the deformed state (as defined in [12]), from black (low strain) to yellow (maximum strain for that column). (Online version in colour.)

Buckling instabilities of elastic structures subjected to deforming forces are found on all scales [1] ranging from large-scale applications such as aircraft to the engineering of DNA [2]. An everyday example can be realized using a plastic coffee stirrer, which deflects laterally if compressed with sufficient force between the forefinger and thumb. As in this example, failure due to compression is typically through a global buckling instability with a wavelength comparable to the length of the structure. Non-uniformities in internal structure introduce localized (short-wave) buckling instabilities that can compete and interact with the global (long-wave) buckling. For example, when under compression the long-wave mode transitions to wrinkling in multilayered composites made of thin interfacial layers embedded in a softer matrix [3]. This short-wavelength buckling behaviour can also be exploited in the design of novel mechanical metamaterials that contain periodic arrays of holes [4]. If an elastic sheet is perforated with a two-dimensional square array of circular holes, the sheet can exhibit pattern switching upon compression that internalizes the buckling: the circular holes deform into ellipses with adjacent holes elongated in orthogonal directions [5,6]. The resulting material properties of the sheet, including negative Poisson’s ratio [7], have been applied to design of photonic [8,9] and phononic [10] cellular devices and have even been used in soft robotics [11]. Similar pattern switching also occurs in a column containing a line of equally spaced holes first studied by Pihler-Puzović *et al.* [12], in which the traditional lateral buckling of a column under compression can be preceded by an instability of the micro-structure between the holes (figure 1).

Here, we focus on in-plane (two-dimensional) buckling of a holey column (figure 1*a*) as an exemplar system to elucidate the mechanisms underlying the pattern switching in perforated elastic solids. As the column is compressed, it initially reduces in length, but preserves its vertical and horizontal reflection symmetries until it reaches a critical strain at which it buckles. We find from numerical calculations that the first unstable mode of in-plane buckling (the mode occurring at the smallest strain) is always one of two modes, which we term the alternating mode and the Euler mode (figure 1*b*), although other modes of buckling, including second- and higher-order Euler modes, bifurcate from the unbuckled compression branch at higher strains. In the alternating mode, the column remains straight under loading and the holes contained within the structure arrange in series of ellipses for which each major axes is orthogonal to its neighbour. The Euler mode is a classic sideways buckling, but, as we shall show, it can act in two qualitatively distinct ways. The Euler mode can act as a *global* mode, where the critical strain and other properties of the mode are dependent on the dimensions of the whole column. It can, alternatively, act as a *localized* buckling mode in which, although deformation occurs throughout the length of the column, the critical strain is independent of column length and depends only on the geometry of the holes.

Pihler-Puzović *et al.* [12] considered a fixed hole size and spacing relative to the column width, identified the buckling modes that occur in two- and three-hole columns, and quantified the transition between the alternating and Euler modes as the length of the column increased. They found good agreement between their finite-element numerical calculations and experiments on holey columns made of the hyperelastic material extra hard Sid AD Special (Feguramed GmbH).

In this paper, we use the validated numerical model from Pihler-Puzović *et al.* [12] to investigate, in detail, the effects of hole size, number and spacing on the pattern formation. We show that the presence of holes in a column introduces localized bucking. This localized buckling occurs not only in the alternating mode but also in the Euler mode, which can be qualitatively altered by the presence of holes. To analyse this localized buckling, we study two-hole columns with periodic boundary conditions, which are unstable only to the localized buckling modes under compression. In addition, we develop a new analytical model for the localized buckling of holey columns when the holes are large and separated by relatively thin ligaments of elastic material. In these columns, the deformation occurs almost entirely through buckling of the ligaments, with thicker regions of the column remaining nearly rigid. This allows the buckling of the column as a whole to be understood through the deformation occurring in the ligaments. The asymptotic predictions from this model have no arbitrary or fitted parameters and compare very favourably with numerical solutions for a surprisingly wide range of hole sizes. Consequently, predictions of the asymptotic behaviour of the system provide insights into the observed pattern formation. In particular, we find that the transition between localized Euler and alternating mode buckling is controlled by the bending stiffness of the two types of ligament in the column: (i) those (initially horizontal) separating the holes and (ii) those (initially vertical) between the hole and the outer edge of the column. We extend the algebraic model to predict the critical stress and strain for deformation of a two-dimensional cellular sheet, which can be viewed as an array of connected holey columns. Good quantitative agreement between these algebraic predictions and numerical finite-element calculations of a cellular sheet provide a simple predictive model of buckling in sheets with square arrays of holes, indicating that the underlying behaviour is also controlled by the ligaments between holes.

The paper is organized as follows: in §2, we use numerical solutions of a plane-strain, finite-element model to study the global and localized buckling of many different holey columns. We briefly revisit the formulation of the problem and the numerical methods from Pihler-Puzović *et al.* [12] in §2a. In §2b, we demonstrate how the Euler mode of buckling is qualitatively altered by the presence of holes, and discuss how the column length and hole geometry result in several different regimes of behaviour that are observed within this mode. In §2c, we show how these geometrical parameters influence buckling in the alternating mode, and in §2d, we describe the ranges of column geometries for which each different mode is the first to become unstable. In §2e, we discuss the secondary bifurcations in the finite-length holey column. Our asymptotic model for a localized buckling column is introduced in §3, and is applied to the alternating mode in §3a and to the Euler mode in §3b. Finally, in §4, we extend the asymptotic model to the two-dimensional cellular sheet. The conclusions are presented in §5.

## 2. Parametric study of Euler and alternating-hole modes

### (a) Numerical model for a finite-length column

We parametrize holey columns of finite length by the number of holes *N*≥2 and the geometry of a repeated ‘unit cell’, described by a width *W*, height *h* and hole diameter *D* (figure 1*a*). The geometry is, therefore, characterized by three non-dimensional parameters: *N*, *D*/*W* and *h*/*W*. As in [12], we investigate the behaviour of the column under compression by solving the equations for an incompressible neo-Hookean^1^ hyperelastic material within this geometry, using the C++ finite-element library oomph-lib [13]. In our numerical code, these equations are formulated using the principle of virtual displacements, with fields discretized within a single finite element using quadratic interpolation for positions and linear interpolation of the solid pressure, which is continuous over element boundaries. We treat the problem as two-dimensional with no out-of-plane buckling and, assuming that the compression is quasi-static, solve for equilibrium states. We also assume that loading by gravity is insignificant.

As the deformations studied here are not large enough to cause contact or self-intersection of the column, we apply zero-stress boundary conditions to the column side walls and the boundaries of the holes. We consider two cases for the boundary conditions at the top and bottom of the column. For columns of finite length, ‘clamped’ boundary conditions are applied, i.e. the top and bottom of the column are constrained to allow no horizontal deformation, and a uniform vertical deformation is prescribed to emulate compression. We also model columns without these boundaries, by applying periodic boundary conditions that match the deformation and stress at the top and bottom edges of a column with two holes. In this case, the distance between the upper and lower boundaries of the periodic domain is varied to apply compression. These periodic columns are not influenced by the presence of upper and lower boundaries, and so they provide a straightforward way of studying localized buckling modes.

We quantify the compression by recording the engineering strain, *ε*=Δ*y*/(*Nh*), where *Nh* is the total length of the uncompressed column and Δ*y* is the amount by which the entire column is compressed vertically. For small strains, and before buckling occurs, the engineering strain varies linearly with engineering stress, defined as the ratio of the total applied compressive force *F* to column width *W*, *E*_eff_*ε*=*F*/*W*. The proportionality constant *E*_eff_ is the effective Young’s modulus. The critical buckling strain, *ε*_*cr*_, is defined as the value of engineering strain at which buckling occurs; the corresponding stress is termed the critical stress. The buckling is a local bifurcation, and therefore occurs at a change in the sign of the real part of the most unstable eigenvalue. We use numerical continuation to solve for both the equilibrium configuration and the stability eigensystem [14], at successively higher compressive strains. A bifurcation tracking procedure is used to determine the compression at which this bifurcation occurs. The domain is discretized for finite element analysis using a mesh that preserves the symmetries of the undeformed configuration, namely the horizontal and vertical reflection symmetries of the column, and the permutation symmetry among the unit cells (figure 1*a*). This choice of mesh facilitates accurate identification of the symmetry-breaking bifurcations. Uniform refinement of the mesh has been used to verify the convergence of the numerical results presented here.

Recall that an ideal undeformed holey column has both horizontal and vertical symmetries. The Euler buckling of a holey column is concurrent with the breaking of the horizontal symmetry (figure 1*b*, centre), and occurs through a pitchfork bifurcation. If the column has an even number of holes, buckling in the alternating mode breaks the vertical symmetry, through a pitchfork bifurcation (figure 1*b*, right). If the column has an odd number of holes (figure 1*c*) the alternating mode buckling does not break the vertical symmetry, and instead occurs via a transcritical bifurcation with two non-conjugate branches that meet in a limit point [12]. We restrict our study to columns with an even number of holes, in which both the Euler mode and the alternating mode are symmetry breaking, each occurring through a supercritical pitchfork bifurcation that breaks a different symmetry. Naturally, the loads at which each of these two bifurcations occurs vary with column length *N* and the geometrical parameters *h*/*W* and *D*/*W*, and the bifurcations may swap their order of occurrence. We now study the behaviour of each mode in turn.

### (b) Euler buckling of perforated columns

**Figure 2.**
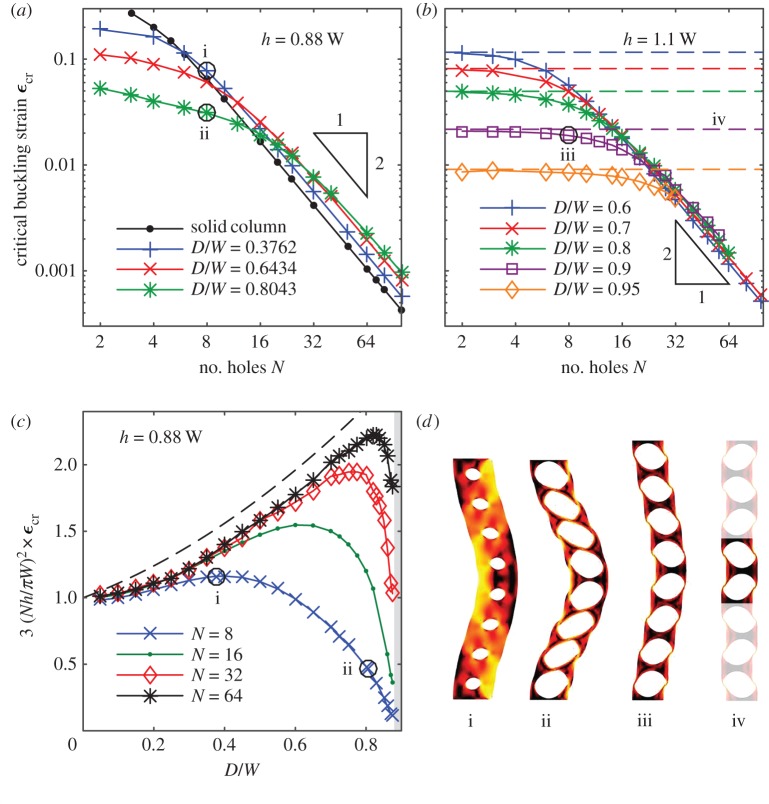
(*a*) Critical buckling strains (*ϵ*_cr_) for the Euler mode, plotted against the number of holes in the column *N* for several hole sizes, for *h*/*W*=0.88. These results are typical of columns in which the distance between hole centres is less than the column width (*h*/*W*<1). Symbols indicate numerical computations, with solid lines added to visualize the trends. The black line indicates numerical results from a solid column (i.e. *D*=0) of length *Nh*. (*b*) Critical buckling strains as in (*a*), but for columns where the distance between hole centres is larger than the column width (*h*/*W*=1.1). Calculations of *ϵ*_cr_ in the localized buckling regime from two-hole periodic columns are shown with dashed lines. (*c*) Critical buckling strains normalized with respect to the critical strain predicted by Euler theory for a solid column of the same width and overall height. Results are shown for *h*/*W*=0.88, though the qualitative features are insensitive to *h*/*W*. The dashed line shows the functional form suggested by [12]. (*d*) Post-buckling states of columns with eight holes after buckling in the Euler mode, with shading indicating local strain energy, increasing from black to yellow, where yellow indicates the maximum local strain energy for each column. Roman numerals identify the bifurcations that led to these states in subfigures (*a*–*c*). Column iv is a periodic column with period of two holes, with the same unit cell geometry and strain as column iii; the periodic extension to eight holes is shown with reduced opacity. (Online version in colour.)

The lateral buckling of long slender columns without holes is described by Euler’s theory, which predicts that the critical strain for buckling scales as the inverse square of the column length [15]. The Euler buckling of sufficiently long columns with a line of holes is qualitatively similar to that of solid columns, with the critical strain for buckling scaling as the inverse square of the column length (or number of holes), *ϵ*_cr_∼1/(*Nh*)^2^ (figure 2*a*–*c*). We call this a global buckling regime, because the critical buckling strain is dependent on the column length, and buckling occurs over the length of the whole column, and not at the lengthscale of the unit cells (figure 2*d*, column i).

Counterintuitively, the critical buckling strain for long columns can be increased beyond the Euler prediction by the addition of holes to the column. This is shown in figure 2*c*, where the critical buckling strain for columns with holes has been normalized with respect to the critical strain predicted by Euler’s theory for a solid column of the same dimensions, and plotted against non-dimensional hole size *D*/*W*. Particularly, for large *N*, the numerical predictions collapse over a range of hole sizes, supporting the scaling *ϵ*_cr_∼1/(*Nh*)^2^, and show a trend of increasing critical strain with increasing hole size. The cause of the increase of critical buckling strain with hole diameter is that enlarging a hole not only decreases the critical buckling stress, but also decreases the effective Young’s modulus of the column. This decrease in Young’s modulus can increase the critical buckling strain of a long column perforated with large holes by a factor of two, relative to the critical buckling strain of a solid column of the same length and width (figure 2*c*). For small holes (*D*/*W*≪1), the critical buckling strain approaches the prediction from Euler’s theory for a solid column, and, during the initial compression phase prior to bifurcation, the stress is concentrated near the holes.

It is evident from figure 2*c* that the collapse of critical buckling strains on a 1/(*Nh*)^2^ scaling no longer holds when the hole size *D*/*W* is large. Geometrical constraints mean that the diameter of the holes *D* must be smaller than both the column width *W* and the distance between hole centres *h*. As the hole diameter approaches this limit, two distinct behaviours are observed in the Euler mode, depending on whether the distance between hole centres is smaller or larger than the column width.

In the first case, when the distance between hole centres is smaller than the column width (*h*<*W*), large holes result in very thin ligaments of elastic material between adjacent holes, in which the strain energy is concentrated. These thin ligaments allow the column to shear with minimal resistance (figure 2*d*, column ii). Consequently, as the hole diameter approaches the distance between hole centres, the critical buckling strain decreases rapidly (figure 2*c*). This reduction in critical buckling strain occurs for a wide range of hole sizes in short columns (N≲8), but is significant in long columns only as the diameter of holes approaches their spacing (*D* approaches *h*).

In the second case, when the distance between hole centres is larger than the column width (*h*>*W*), large holes instead result in very thin ligaments of elastic material separating the edges of the column from the holes, with relatively thicker regions of material between adjacent holes. In contrast with the behaviour when *h*<*W*, the critical buckling strain of such columns is nearly independent of the number of holes (figure 2*b*), and decreases with increasing hole size. The buckling of the whole column in this regime results from the individual buckling of the thin ligaments at the edges of the column (figure 2*d*, column iii). We refer to this as the ‘sliding’ regime of the Euler mode, as the thicker regions of the column separating the holes do not deform or rotate significantly, but instead slide left or right. An increase in the hole diameter *D* reduces the thickness of the ligaments at the edges of the column and causes a corresponding decrease in the critical buckling strain (figure 2*b*). The buckling of the ligaments at the edges of the column in this sliding regime is in contrast with the global regime of buckling (*ϵ*_cr_∼1/(*Nh*)^2^) observed for long columns, in which the ligaments at the edges of the column are compressed or stretched by the bending of the column, but do not individually buckle.

As the critical buckling strain for the column in the sliding regime is independent of the number of holes in the column, it depends only on the localized geometry of these thin ligaments. In the sliding regime, the deformation of each ligament is isolated from the deformation in adjoining ligaments by the thicker region of material between holes. This means the ligaments at the edges of the column buckle to the left or to the right almost independently of one another. A consequence of this is that the critical buckling strain in this sliding regime is in quantitative agreement with the buckling strain in a two-hole periodic column with the same unit cell geometry (dashed lines in figure 2*b*), in which the ligaments buckle alternately left and right (figure 2*d*, column iv). A related consequence of this isolation of the buckling in adjacent holes is that the critical buckling strains for second- and higher-order Euler modes in the sliding regime are only slightly larger than that of the first Euler mode. This ‘clustering’ of many successive Euler modes around a single critical strain in the sliding regime explains why the two-hole periodic column is a good predictor of the critical strain of the first Euler mode (figure 2*d*, column iii), despite the fact that when extended periodically to *N* holes it is closest in appearance to the Euler mode of order (*N*−1) (figure 2*d*, column iv).

The addition of a line of holes to an elastic column, therefore, significantly alters its Euler buckling behaviour. The main influence of the holes is the new sliding regime of buckling, which is a localized buckling mode that depends on the properties of the holes and not the column length. Importantly, this sliding regime is not a separate mode of instability, but is a new regime of behaviour of the existing Euler buckling mode, induced by the presence of holes. There is consequently a continuous transition between the localized behaviour of the sliding regime and the classical Euler buckling regimes, as illustrated in figure 2*b*.

### (c) Alternating mode buckling

**Figure 3.**
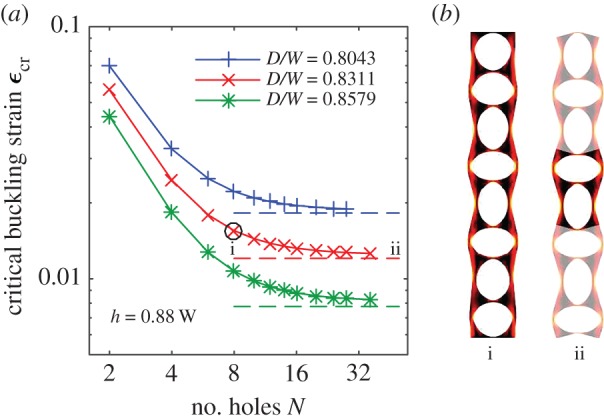
(*a*) Critical buckling strains (*ϵ*_cr_) for the alternating mode, plotted against column length (*N*, number of holes) for several hole sizes. Symbols indicate numerical computations, with solid lines added to visualize the trends. (*b*) (i) Post-buckling state of a column with *N*=8, *D*/*W*=0.8311 after buckling in the alternating mode, with shading indicating local strain energy, increasing from black to yellow. (ii) A two-hole periodic column with the same unit cell geometry and strain as column i; the periodic extension to eight holes is shown with reduced opacity. (Online version in colour.)

The critical buckling strain for the alternating mode is qualitatively different from the Euler mode, in that it tends to be a constant for long columns (figure 3*a*), rather than scaling as 1/*N*^2^. The critical buckling strain for long columns is in good agreement with that of a two-hole periodic column buckling in the alternating mode, indicated by dashed lines in figure 3*a*. As with the sliding regime of the Euler mode, this is a localized buckling mode, characterized by independence of the critical strain on *N*, and agreement between predictions from finite-length and periodic columns. The post-bucking states, illustrated in figure 3*b*, indicate that buckling of both the ligaments separating holes and the ligaments on the column edge occurs in this localized mode. The critical buckling strain for the alternating mode decreases with increasing hole size *D*/*W*, the opposite trend from that of long columns in the Euler buckling mode.

In the alternating mode, the critical strain in short columns is larger than that in long columns. This increase in strain is caused by boundary effects from the clamped boundary conditions; these boundary conditions prevent bending of the ligaments at the top and bottom of the column, and so prevent the alternating mode in finite columns from being exactly periodic. This behaviour can be seen in figure 3*b*, where the buckling of the finite-length column (column i) is close to periodic (column ii) everywhere except near the top and bottom boundaries.

### (d) Exchange of stability between Euler and alternating modes

The number of holes *N* and the geometrical parameters *h*/*W* and *D*/*W* determine which of the two modes, Euler or alternating, occurs at the onset of the instability from the unbuckled compression branch.

**Figure 4.**
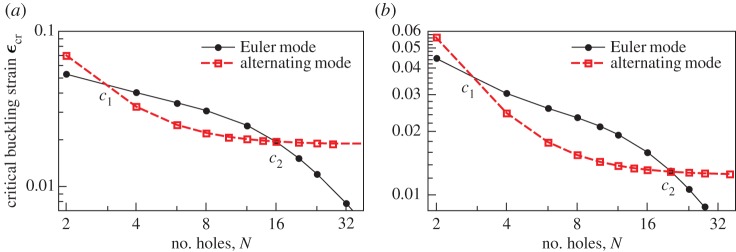
Critical strains for the Euler and alternating modes compared, for *h*/*W*= 0.88 and hole sizes (*a*) *D*/*W*=0.8043 and (*b*) *D*/*W*=0.8311, as in figures 2*a* and 3*a*. For columns with a number of holes *N* between *c*_1_ and *c*_2_, the alternating mode becomes unstable at a lower strain than the Euler mode, and is thus the mode that would be observed experimentally. The Euler mode occurs at a lower strain when *N*<*c*_1_ or *N*>*c*_2_. Lines between the discrete points are added to guide the eye. (Online version in colour.)

The critical strain at which buckling occurs in the Euler mode and the critical strain at which buckling occurs in the alternating mode are compared in figure 4 as functions of the number of holes in the column *N*. At large *N*, the *ϵ*_cr_∼1/*N*^2^ behaviour of the Euler mode and *ϵ*_cr_∼*const*. behaviour of the alternating mode means that sufficiently long columns will always buckle first in the Euler mode. For short columns, the frustration of the alternating mode by the clamped boundary conditions (leading to increased *ϵ*_cr_) has the same result; sufficiently short columns also buckle in the Euler mode. For the shortest column with an even number of holes, *N*=2, we have found no column geometry where the alternating mode bifurcation (figure 1*b* right) occurs at a lower strain than the Euler mode (figure 1*b* centre). However, at intermediate column lengths, the first buckling instability of the column can be in the alternating mode.

**Figure 5.**
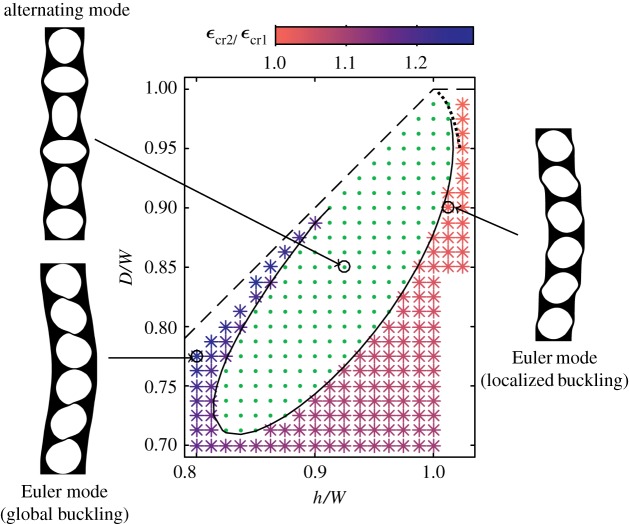
Numerical calculation of the first-occurring mode for a column with *N*=6 holes, as a function of the geometrical parameters of non-dimensional hole size and hole spacing. All solutions lie below the dashed line, which indicates the largest possible hole size *D*/*W*. The solid black line indicates parameters in which the alternating and Euler-type modes occur at the same critical strain. This line divides the parameter space into a regions in which the alternating mode (dots) or Euler mode (stars) occur at the smallest strain. The ratio of critical strains for the onset of the first and second Euler buckling mode *ϵ*_*cr*2_/*ϵ*_*cr*1_ is indicated by the colour of the stars. The dotted black line is an asymptotic prediction for the boundary between alternating and Euler mode in the localized/sliding regime (3.23). The three solutions illustrated are post-buckling solutions on the primary branch originating from the first bifurcation to occur at each of the highlighted points. (Online version in colour.)

The first mode to become unstable is illustrated for such a column of intermediate length (*N*=6 holes) in figure 5, as a function of both hole size *D*/*W* and hole spacing *h*/*W*. Each point on the figure corresponds to a numerical computation of solutions along the unbuckled compression branch, in which the critical strains at which the Euler and alternating bifurcations occur are recorded. Crosses show the region in parameter space where the Euler mode bifurcation occurs at a lower strain than the alternating mode bifurcation, and dots the region where the alternating mode occurs at a lower strain. The solid black line separating these two regions indicates the column geometries for which the two bifurcations occur at the same strain, and is obtained by plotting the zero contour of the difference between the critical strains for the Euler and alternating bifurcations. The alternating mode occurs at a lower strain than the Euler mode in regions of this geometric parameter space where the ligaments are relatively thin both at the side walls of the column and between the holes. In other regions, the Euler mode occurs at a lower strain.

Comparison of figure 2*a* and 2*b* suggests that an increase in *h*/*W* promotes the localized sliding regime of the Euler mode. This is supported by the results in figure 5, in which the colours of the crosses indicate the ratio between critical strains of the second to first Euler mode bifurcations; as noted previously, these occur at very similar strains in the localized sliding regime. For a given hole size *D*, figure 5 indicates that the alternating mode is preferred for a limited range of hole spacings *h*/*W*. For holes spaced more widely than this range, the ligaments separating holes are thick, and a localized Euler mode in the sliding regime is preferred, where these ligaments separating holes do not deform significantly. For holes spaced more closely than this range, the ligaments at the edge of the column are thick compared to those separating holes, and a global Euler buckling mode is preferred, in which the thicker ligaments adjoining the edge of the column do not buckle, except over the length of the whole column.

Our simulations suggest that as the column length increases, the region of the alternating mode in the phase space of the unit cell geometry (dots in figure 5) shrinks and ultimately disappears at around *N*=16 holes. In a sufficiently long column, the first bifurcation to occur during compression, therefore, always corresponds to the Euler mode. However, the alternating mode may still play a role in the subsequent deformation, due to the presence of secondary bifurcations.

### (e) Secondary bifurcations

Our study, so far, has explored states arising at the primary bifurcation that occurs at the smallest strain on the unbuckled compression branch. We now study the secondary bifurcations that occur on the first branch to bifurcate from the unbuckled compression branch, either the alternating mode or the first Euler mode.

**Figure 6.**
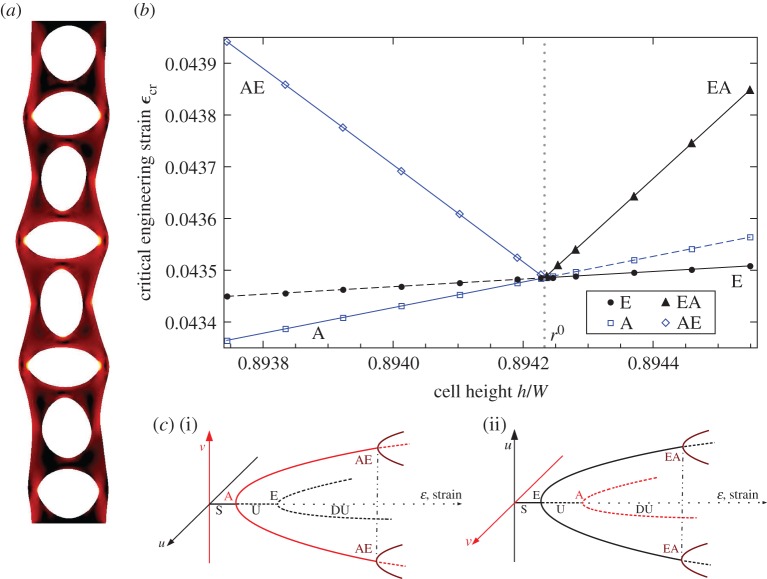
(*a*) Eigenmode of a secondary bifurcation in an eight-cell-long holey column made from cells with aspect ratio *h*/*W*=0.88 and hole size *D*/*W*=0.7525. Shading indicates strain energy as in figure 1. (*b*) Critical buckling strains *ϵ*_cr_ plotted against cell height for eight-cell-long holey columns with *D*/*W*=0.7525, for Euler mode (E), alternating mode (A) and secondary bifurcations (AE and EA, arising on the primary alternating mode and the primary Euler mode branches, respectively). The lines between the discrete points are added to guide the eye and mark bifurcations that occur on stable branches (solid lines) and on unstable branches (dashed lines). (*c*) Schematic bifurcation diagrams for two different scenarios separated by the critical aspect ratio (*h*/*W*≡*r*^0^=0.89426). When *h*/*W*>*r*^0^ (ii), E and EA modes are stable. (i) When *h*/*W*<*r*^0^, the A and AE modes are stable and E is unstable. (ii) When *h*/*W*>*r*^0^, the E and EA modes are stable, whereas A becomes unstable. (Online version in colour.)

In both cases, the primary bifurcation at the onset of the instability is supercritical. The continued compression of the column, therefore, initially results in stable solutions with a single broken symmetry that have bifurcated from the unbuckled compression branch. On these already bifurcated solution branches, we find that secondary bifurcations can exist, of the type first described by Bauer *et al.* [16]. These secondary bifurcations result in solutions with a second broken symmetry; in the example shown in figure 6*a*, a column with eight holes exhibits both the lateral buckling and the alternating mode, with both horizontal and vertical symmetries broken.

We examine the behaviour of secondary bifurcations in the neighbourhood of a point in the geometric parameter space at which the Euler and alternating modes bifurcate from the unbuckled compression branch at the same critical strain. Choosing *N*=8 and *D*/*W*=0.7525, this point occurs at *h*/*W*=*r*^0^≈0.89426, and is marked by the vertical dashed line in figure 6*b* that separates the two regions of parameter space, in which either the Euler mode (*h*/*W*>*r*^0^) or the alternating mode (*h*/*W*<*r*^0^) occurs at the lowest strain on the trivial branch. By exploring the secondary bifurcations, we investigate the nature of the stability exchange between the two modes.

In numerical calculations, we impose increasing applied strain until we reach the first bifurcation to occur on the unbuckled compression branch, at which point we perturb the numerical solution with the eigenvector associated with the bifurcation. This bifurcation is supercritical, so by increasing the applied strain further we find solutions on the bifurcated branch until the secondary bifurcation is located. The critical strains of both the primary and secondary bifurcations are shown in figure 6*b*. For *h*/*W*<*r*^0^, the alternating mode bifurcation (A) occurs at the lowest strain, and the solutions with broken top–bottom symmetry that originate from this bifurcation are subject to the secondary bifurcation (AE) where the solutions also buckle laterally, breaking the left–right symmetry (figure 6*c*). For *h*/*W*>*r*^0^, order of the bifurcations is reversed: here the Euler mode bifurcation (E) occurs at the lowest strain, and the laterally buckled solutions that originate from this bifurcation subsequently become unstable to an alternating mode (breaking the top–bottom symmetry) at the secondary bifurcation (EA). At *h*/*W*=*r*^0^, the Euler and alternating mode primary bifurcations occur at the same strain, and all the bifurcation points, both primary (A and E) and secondary (AE and EA), then coalesce to meet at the same critical strain, forming a multiple primary bifurcation point on the unbuckled compression branch. This feature has been identified and explained by previous studies on secondary bifurcations [16,17]. The coalescence of critical strains of the primary and secondary bifurcations gives insight into the behaviour of columns when they are compressed beyond the initial instability; for columns with geometry where the alternating and Euler modes occur at nearly the same critical strain (i.e. those close to the black line in figure 5), only a small additional compression is required for both top–bottom and left–right symmetries of the column to be broken.

## 3. Theoretical modelling of localized buckling modes

**Figure 7.**
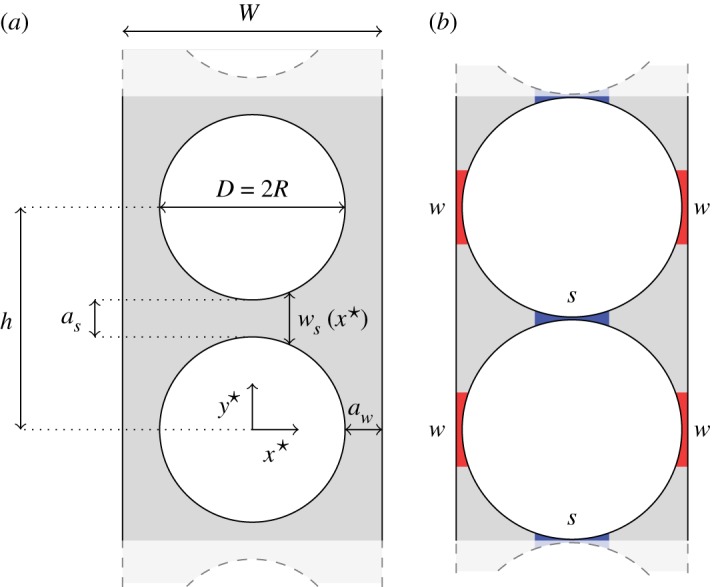
(*a*) Definitions of the width of column *W*, hole spacing *h*, minimum thickness of side-walls *a*_*w*_ and minimum width of gap between holes *a*_*s*_ in a two-hole periodic unit cell. (*b*) Thin ligaments adjoining the column edge (shaded red) are labelled *w*, and ligaments separating two holes (shaded blue) are labelled *s*. (Online version in colour.)

Motivated by the existence of localized buckling modes and their importance in the overall behaviour of the column, we develop a theoretical model for the stress at the onset of bifurcation in a short section of column with two holes and with periodic boundary conditions imposed on the top and bottom edges (figure 7*a*). These periodic columns with a period of two holes exhibit instability to both the alternating mode (figure 3*b*, column ii) and to the localized sliding regime of the Euler mode (figure 2*d*, column iv). Furthermore, as we have shown, the critical buckling strains of the periodic columns are in quantitative agreement with the appropriate localized buckling regime of non-periodic columns of finite length: localized Euler buckling in short columns with hole spacing larger than the width (figure 2*b*), and alternating mode buckling in long columns (figure 3*a*). The theory presented in this section is, thus, applicable both to periodic columns and to the localized buckling regimes of non-periodic columns, where the buckling properties are independent of the column length.

When the holes in the column are similar in diameter to the column width or hole spacing, the deformation is localized, occurring through bending of the resulting thin regions of elastic material, or ligaments, highlighted in figure 7*b*. The thicker uncoloured parts of the structure remain comparatively rigid. By exploiting the large aspect ratio of the ligaments, and the comparative rigidity of other parts of the structure, we can obtain algebraic expressions for the critical buckling force for each mode. There are two types of ligament present in the system; those separating two holes, which we denote *s*, and those adjoining the side wall of the column, which we denote *w* (figure 7*b*). The minimum width of these ligaments is

3.1 $$a_{s}=h-D\;\mathrm{and}\;a_{w}=\frac{1}{2}(W-D),$$

respectively, and we develop the model in the asymptotic limit where *a*_*s*_≪*D* and *a*_*w*_≪*D*.

The width of the ligament separating two adjacent holes *w*_*s*_ (figure 7*a*) is given as a function of the coordinate along its length *x*^⋆^ by

3.2 $$w_{s}=2\left(\frac{a_{s}}{2}+R-\sqrt{R^{2}-x^{⋆2}}\right)=a_{s}\left(1+\frac{x^{⋆2}}{Ra_{s}}+\cdots \right),$$

where *R*=*D*/2 is the radius of the hole. As expected from the geometry in figure 7*a*, *w*_*s*_ takes a minimum value *a*_*s*_ when *x*^⋆^=0. Similarly the width *w*_*w*_ of a ligament adjacent to the edge of the column, this time dependent on the coordinate *y*^⋆^, is

3.3 $$w_{w}=a_{w}\left(1+\frac{y^{⋆2}}{2Ra_{w}}+\cdots \right).$$

When a ligament is of a similar thickness to its thinnest point (*w*_*s*_=*O*(*a*_*s*_)), we find from (3.2) that the ligament has lengthscale $x^{⋆}=O(\sqrt{Ra_{s}})\gg O(a_{s})$, implying that the ligament is much longer than it is wide. We can, therefore, model the ligaments as thin Euler–Bernoulli beams of non-uniform width, with the width of the two types of ligament given by (3.2) and (3.3).

### (a) Alternating mode

**Figure 8.**
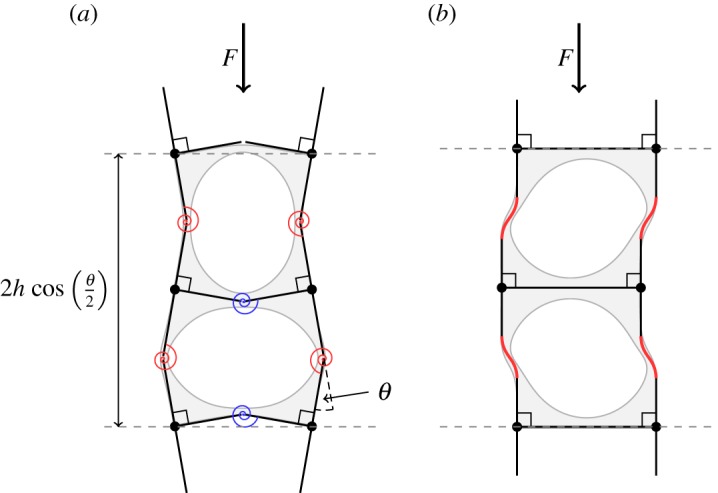
Sketches of the asymptotic models for (*a*) the alternating mode and (*b*) the Euler mode in the localized sliding regime, for a two-hole periodic column. Finite-element calculations of post-buckling states, in light grey, are overlaid with sketches indicating the deformation of the thin ligaments and the parts of the structure that we assume are rigid when the holes are large. (*a*) The torsion spring model for the alternating mode bifurcation. The springs illustrated (arising from four *w* ligaments and two *s* ligaments) are those in a single instance of the two-hole periodic cell. The applied force *F* is exerted at the centre of each rotating T-shaped element (marked by a black-filled circle). Each torsion spring is bent by an angle *θ*. (*b*) The Euler mode bifurcation in a two-hole periodic unit cell, in the localized sliding regime. The thin *s* regions separating the holes do not deform in this mode, but buckling occurs in the four thin *w* regions adjacent to the edges of each periodic cell. (Online version in colour.)

Motivated by the results of finite-element computations, which indicate that when *a*_*s*_,*a*_*w*_≪*R*, the strain energy is concentrated almost entirely in the thin ligaments, we assume that a column buckling in the alternating mode can be modelled as a system of rigid sections, formed from thick regions of elastic material, that are connected by the thin, flexible ligaments. Each ligament acts as a hinge with a torsion spring (figure 8*a*).

We start by calculating the stiffness of these torsion springs from the ligament geometry, using Euler–Bernoulli beam theory. The equation for small deformations of an Euler–Bernoulli beam [15] is

3.4 $$\frac{d^{2}}{dx^{2}}\left(EI\frac{d^{2}\phi }{dx^{2}}\right)=0,$$

where *E* is Young’s modulus of the material, *x* is the distance along the beam, *ϕ* is the perpendicular displacement of the beam,

3.5 $$I=\int_{-w/2}^{w/2}y^{2}\;dy=\frac{w^{3}}{12}$$

is the second moment of the cross-sectional area of the beam, and *w*(*x*) is the width of the beam, given for the two types of ligament by (3.2) and (3.3). Integrating (3.4) twice gives

3.6 $$EI\frac{d^{2}\phi }{dx^{2}}=M,$$

where *M* is the bending moment. As the length of each ligament is much smaller than the column width or hole spacing, the moment *M* is constant (to leading order) along the length of the ligament. We consider a ligament with a general parabolic width profile

3.7 $$w=a\left(1+\frac{x^{2}}{x_{0}^{2}}\right),$$

where the constants *a* and *x*_0_ parametrize the beam geometry in either type of ligament (see (3.2), (3.3)). Using (3.7) in (3.5) reduces (3.6) to

3.8 $$\frac{d^{2}\phi }{dx^{2}}=\frac{12}{Ea^{3}}\frac{M}{{\left(1+x^{2}/x_{0}^{2}\right)}^{3}}.$$

Integrating again and seeking a symmetric solution with d*ϕ*/d*x*=0 at *x*=0, we obtain

3.9 $$\frac{d\phi }{dx}=\frac{3Mx_{0}}{2Ea^{3}}\left(\frac{X(5+3X^{2})}{{\left(1+X^{2}\right)}^{2}}+3\;\tan^{-1}X\right),$$

where *X*=*x*/*x*_0_. We note that

3.10 $$\lim_{x\to \pm \infty }\frac{d\phi }{dx}=\pm \frac{9\pi }{4}\frac{Mx_{0}}{Ea^{3}},$$

namely that the beam becomes straight at large |*x*|. For small deflections of the beam, we may, therefore, define a bending angle for the entire beam, as

3.11 $$\theta =\lim_{x\to \infty }\frac{d\phi }{dx}-\lim_{x\to -\infty }\frac{d\phi }{dx}.$$

From (3.10), the moment exerted by the beam can be written as

3.12 M=κθ,

where torsion coefficient *κ* is

3.13 $$\kappa =\frac{2Ea^{3}}{9\pi x_{0}}.$$

Setting *a*=*a*_*s*_ and $x_{0}=\sqrt{Ra_{s}}$ for the *s* ligaments (3.2), and *a*=*a*_*w*_ and $x_{0}=\sqrt{2Ra_{w}}$ for the *w* ligaments (3.3), we obtain the expressions for torsion coefficient of the spring formed by these ligaments as

3.14 $$\kappa_{s}=\frac{2}{9\pi }E{\left(\frac{a_{s}^{5}}{R}\right)}^{1/2}\;\mathrm{and}\;\kappa_{w}=\frac{\sqrt{2}}{9\pi }E{\left(\frac{a_{w}^{5}}{R}\right)}^{1/2},$$

respectively. With these coefficients evaluated, we can now use (3.12) to relate the angle by which each ligament is bent, *θ*, to the moment it exerts *M*.

A single periodic unit of our model of the holey column consists of four *w* ligaments and two *s* ligaments (figure 8*a*). When this periodic unit is compressed by a force *F* and each of these ligaments is bent by an angle *θ*, the height of the periodic unit is reduced from 2*h* to 2hcos⁡(θ/2). The potential energy of this unit is, therefore,

3.15 $$V=(2\kappa_{s}+4\kappa_{w})\frac{1}{2}\theta^{2}+2h\;\left[\cos\left(\frac{\theta }{2}\right)-1\right]F$$

and the equilibria lie at the stationary points of this energy, where

3.16 $$\frac{\partial V}{\partial \theta }=(2\kappa_{s}+4\kappa_{w})\theta -hF\;\sin\;\left(\frac{\theta }{2}\right)=0.$$

The change in stability of the equilibrium branch at *θ*=0, a bifurcation, occurs at a critical force,

3.17 $$F=\frac{1}{h}(4\kappa_{s}+8\kappa_{w})=\frac{8}{9\pi }E\frac{a_{s}^{5/2}+\sqrt{2}a_{w}^{5/2}}{h\sqrt{R}}.$$

**Figure 9.**
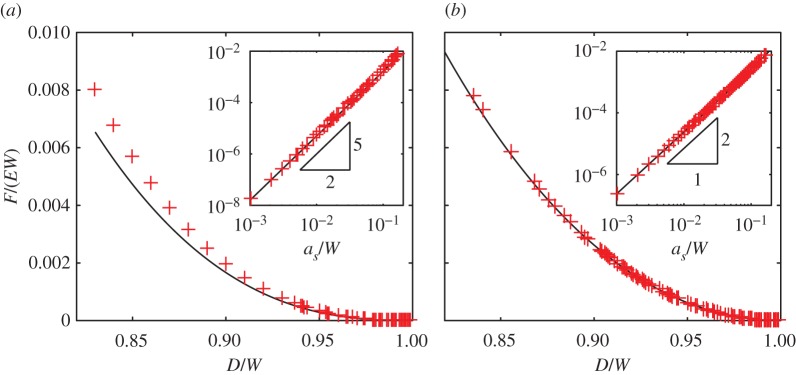
The force at the onset of (*a*) the alternating mode, and (*b*) the Euler mode in the localized sliding regime, for periodic columns with a square unit cell, *h*=*W*. Asymptotic predictions (black curves), equations (3.17) and (3.22) are compared with finite-element numerical calculations (red crosses). Insets: the same data plotted on log–log axes in terms of a non-dimensional ligament thickness *a*_*s*_/*W*, illustrating that for thin ligaments, the force scales as the ligament thickness to the power 5/2 for the alternating mode (3.17) and power 2 for the Euler mode (3.22). (Online version in colour.)

To validate this prediction, we compare it to the critical force at the bifurcation obtained from finite-element numerical calculations of a two-hole column with periodic boundary conditions applied to the top and bottom of the column. The compression of the system is controlled by altering the vertical offset between periodic units in the deformed configuration, and the force *F* measured by integrating the traction over the upper boundary. For these computations, an unstructured triangle mesh was used to discretize the domain, with elements concentrated in the thin ligaments. Our theoretical prediction (3.17) closely matches the numerical calculations, particularly as the hole diameter approaches the width of the column (figure 9*a*).

### (b) Localized Euler buckling

Euler buckling of the two-hole periodic system occurs in the localized sliding regime. In this mode, only the *w* ligaments adjoining the edges of the column undergo deformation, whereas the *s* ligaments separating holes are undeformed (figure 8*b*). Here, we derive the critical force required for this Euler mode to be realized.

The buckling analysis for this problem is very similar to that for classical Euler buckling (e.g. [18]). Each *w* ligament supports half the compression force *F*, and so from (3.6) we have

3.18 $$EI\frac{d^{2}\phi }{dx^{2}}=-\frac{F}{2}\phi ,$$

where the moment is the product of the force *F*/2 and the perpendicular distance *ϕ*. Evaluating *I* using the geometrical parameters corresponding to the *w* ligaments (3.3), as in (3.8), gives

3.19 $${\left[1+X^{2}\right]}^{3}\frac{d^{2}\phi }{dX^{2}}=-\frac{12RF}{Ea_{w}^{2}}\phi ,$$

where $X=x/\sqrt{2Ra_{w}}$. In the sliding regime of the Euler mode, the thicker regions of the beam between ligaments do not rotate, and so the boundary conditions are ‘clamped’, but able to slide in a direction perpendicular to the load,

3.20 $$\frac{d\phi }{dX}\to 0\;\mathrm{as}\;X\to \pm \infty .$$

Seeking non-zero solutions to the Sturm–Liouville boundary value problem (3.19)–(3.20), we obtain a discrete spectrum of eigenmodes, and find numerically the smallest eigenvalue,

3.21 $$\frac{12RF}{Ea_{w}^{2}}=5.668\ldots ,$$

which corresponds to an odd mode (*ϕ*(*X*)=−*ϕ*(−*X*)). The critical force for bifurcation is then

3.22 $$F\approx 0.472\frac{Ea_{w}^{2}}{R}.$$

The theoretical expression (3.22) agrees extremely well with the results obtained from finite-element calculations, even when *a*_*w*_/*R* is relatively large (figure 9*b*). As the thin *s* ligaments separating adjacent holes do not deform in this mode, the critical stress (3.22) is independent of *a*_*s*_, and the calculation of this critical stress does not require that *a*_*s*_/*R*≪1.

Comparing our theoretical predictions for the forces for the onset of the alternating and Euler modes, (3.17) and (3.22), we find that the alternating mode bifurcation occurs at a lower force than the Euler bifurcation when

3.23 $$\left[{\left(\frac{a_{s}}{a_{w}}\right)}^{5/2}+\sqrt{2}\right]\frac{\sqrt{Ra_{w}}}{h}<1.67.$$

**Figure 10.**
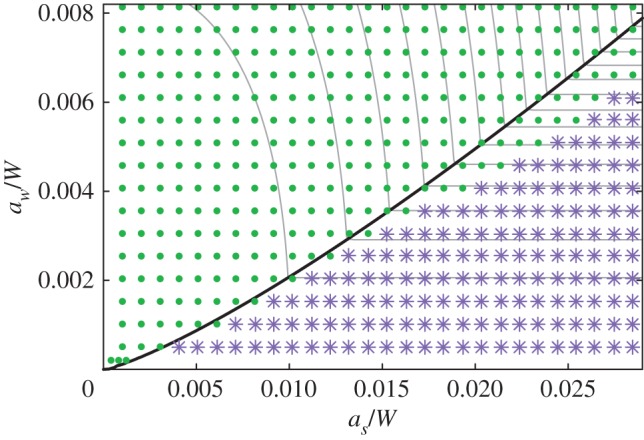
The scatter points indicate finite-element calculations of the first buckling mode: solid circles denote parameters where the alternating bifurcation occurred at a lower critical force compared to the Euler mode, and stars denote the opposite. The theoretically predicted boundary between these regimes (3.23) is plotted in thick black line; grey lines are contours of the critical for in each mode, from (3.17) and (3.22). (Online version in colour.)

As illustrated in figure 8, this inequality expresses the conditions under which bending of four *w* ligaments and two *s* ligaments (in the alternating mode) occurs at a lower critical strain than buckling of the four *w* ligaments (in the Euler mode). This prediction is in agreement with numerical calculations of the critical buckling of periodic columns, particularly when *a*_*w*_,*a*_*s*_≪1 (figure 10). The criterion (3.23), plotted as a dotted line in figure 5, is also in general agreement with numerical calculations of finite-length columns in the region of parameter space where both alternating and Euler modes are localized and so are well predicted by the results from periodic columns.

## 4. Asymptotic model for the onset of bifurcation in a two-dimensional cellular sheet

**Figure 11.**
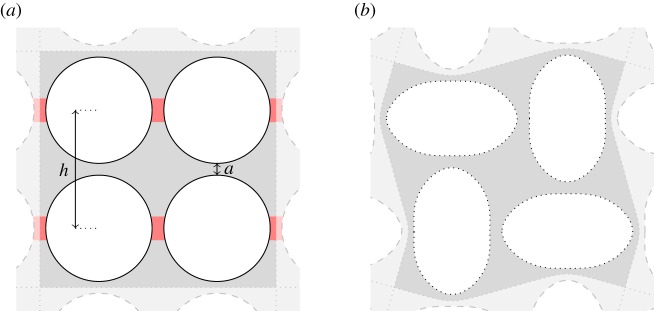
(*a*) Sketch of a single periodic unit of the two-dimensional perforated sheet with *a*/*h*=0.1. The red regions indicate the thin regions that undergo compression prior to the onset of the bifurcation. (*b*) Configuration of the same perforated sheet after buckling, obtained from finite-element computation. The periodic unit of initial size 1×1 has been compressed to a height of 0.953 and its width has reduced (due to the auxetic behaviour of the sheet) to 0.977. (Online version in colour.)

The asymptotic theory for bifurcation presented in the previous section extends naturally to two-dimensional cellular solids perforated by a square lattice of circular voids, where the geometry is described by a single non-dimensional parameter, *a*/*h*, representing the minimum distance between holes divided by the spacing between hole centres (figure 11). When compressed uniaxially, such two-dimensional cellular materials exhibit an alternating-mode bifurcation at a critical strain [5,7], similar to the bifurcation we have demonstrated in columns. Changing the pattern of holes in the solid, for example to a triangular lattice, leads to a range of related instabilities [19], which can be exploited in the fabrication of metamaterials with desired mechanical properties [4]. As before, we consider only two-dimensional buckling modes, neglecting out-of-plane buckling, which may occur in sufficiently thin plates (e.g. [20]).

The onset of the buckling instability in cellular sheets has been studied numerically, under general in-plane loading in the case where *a*/*h*=1/2 [21], and under uniaxial compression for more general *a*/*h* [7]. Here, we derive algebraically how the critical force *F*_cr_ and strain *ϵ*_cr_ vary with the hole size *a*/*h*, under uniaxial compression of the solid, when the holes are relatively large compared with the elastic regions separating them (*a*/*h*≪1).

Our model for alternating-mode buckling in elastic cellular sheets perforated with large holes is similar to that in holey columns (figure 8*a*), in that we assume that the thin ligaments bend, while thicker regions of the structure rotate without significant deformation. This approach is supported by the concentration of the strain energy within the ligaments in numerical calculations. Furthermore, the approach predicts that Poisson’s ratio of the cellular sheet approaches −1 for large holes, which is consistent with numerical calculations [7].

Defining the hole radius as *R*=(*h*−*a*)/2, we find from (3.13) that the bending strength of a single ligament is

4.1 $$\kappa =\frac{2}{9\pi }E\;{\left(\frac{a^{5}}{R}\right)}^{1/2}.$$

A single periodic cell of two-by-two holes contains eight such ligaments, so the potential energy of a single periodic cell of this system with an applied compression force *F* is

4.2 $$V=8\cdot \frac{1}{2}\kappa \theta^{2}+2hF\;\left[\cos\left(\frac{\theta }{2}\right)-1\right].$$

As before, the equilibria lie at the stationary points of the energy,

4.3 $$\frac{\partial V}{\partial \theta }=8\kappa \theta -hF\;\sin\;\left(\frac{\theta }{2}\right)=0$$

and a bifurcation on the solution branch *θ*=0 thus occurs at at a critical force *F*_cr_, where

4.4 $$\frac{F_{\mathrm{cr}}}{E}=\frac{16\kappa }{h}=\frac{32}{9\pi h}{\left(\frac{a^{5}}{R}\right)}^{1/2}.$$

In addition to this critical force, the symmetry of the two-dimensional lattice also allows us to calculate easily the critical strain at bifurcation. We do this by evaluating the effective Young’s modulus of the cellular solid under small loads, before the bifurcation occurs. The solid can be divided into two regions: a region of length and width *O*(*h*) (shaded grey in figure 11*a*), and a region corresponding to thin ligaments that are parallel to the direction of compression, of length *O*((*aR*)^1/2^) and width *O*(*a*) (shaded red in figure 11). The linear deformations resulting from applying a force to these regions scale^2^ as the length of the region (in the direction of applied stress) over its width. The deformation in the two regions of the holey sheet, therefore, scales as *h*/*h*=*O*(1) for the thick (grey shaded) regions and as *O*((*R*/*a*)^1/2^)≫1 for the thin (red shaded) parts. These scalings suggest that, for small deformation when *a*/*h*≪1, the thick regions may be considered rigid and the deformation of the whole periodic unit predominantly results from compression of the thin ligaments that are parallel to the applied strain.

Each periodic repeating unit of the solid consists of four such ligaments, each of which supports a force *F*/2. When compressed under a force *F*/2, the length change of a single column of the two-dimensional lattice is

4.5 $$\begin{array}{rl} \Delta l & =\frac{F}{2E}\int_{-\infty }^{\infty }\frac{1}{a[1+{\left(x/\sqrt{Ra}\right)}^{2}]}\;dx \end{array}$$

4.6 $$\begin{array}{r} =\frac{\pi F}{2E}{\left(\frac{R}{a}\right)}^{1/2}. \end{array}$$

The total compression of the repeating periodic unit is given by 2Δ*l*, and so the relative strain is

4.7 $$\epsilon_{\mathrm{cr}}=\frac{2\Delta l}{2h}=\frac{\pi }{2Eh}{\left(\frac{R}{a}\right)}^{1/2}F.$$

This relationship between *ϵ*_cr_ and *F*, which holds immediately prior to a bifurcation, allows us to convert the critical force for the bifurcation (4.4) into a critical strain,

4.8 $$\epsilon_{\mathrm{cr}}=\frac{16}{9}{\left(\frac{a}{h}\right)}^{2}.$$

**Figure 12.**
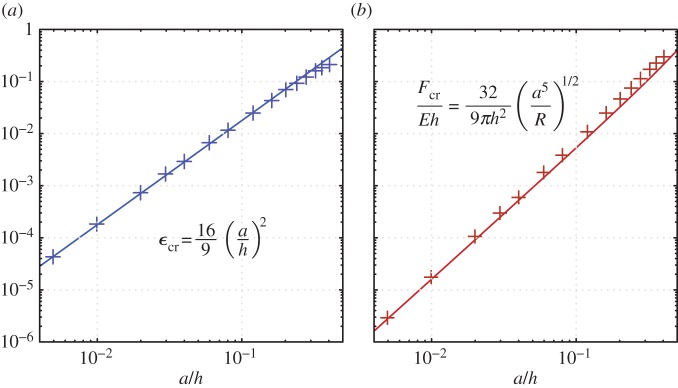
(*a*) Critical strain *ϵ*_cr_ and (*b*) non-dimensional critical force *F*_cr_/(*Eh*) at the bifurcation of a two-dimensional cellular sheet, obtained from asymptotic theory (solid line) and numerical simulation (crosses). (Online version in colour.)

The asymptotic calculations for the critical force (4.4) and strain (4.8) are in good agreement with numerical computations of the compression of a neo-Hookean material for thin-walled two-dimensional lattices (figure 12). In these computations, we simulate a single periodic unit of four holes, with periodic boundary conditions applied on both the upper/lower and left/right boundaries. As with the periodic columns, the amount of compression is imposed by specifying the periodic offset in the vertical direction. At the left and right periodic boundaries, we leave the horizontal periodic offset undetermined, and instead specify that the integral of the stress over the sides of the periodic unit is zero. The periodic cell is, therefore, free to expand or contract horizontally as it is compressed (figure 11*b*). For *a*/*h*=1/10 (as illustrated in figure 11), the asymptotic prediction of the critical stress (4.4) is within 5% of the value obtained from numerical computations, with closer agreement at smaller values of *a*/*h*. Our asymptotic prediction from (4.8) of *ϵ*_cr_=0.03 for *a*/*h*=0.13 is also in good agreement with the change in stress–strain behaviour observed in the periodic numerical calculations of Mullin *et al.* [5], and is similar to their experimental measurement of *ϵ*_cr_=0.04 for a sheet with 10×10 holes.

## 5. Conclusion

We have used a previously validated plane-strain, finite-element model [12] to investigate the in-plane buckling behaviour of a holey column under uniaxial compression, focusing on how this behaviour varies with the column geometry; specifically the number, size and spacing of the holes. Insight into the detailed mechanics of buckling is provided by a new asymptotic theory, which quantitatively predicts the critical stress at which a column with large holes buckles, without arbitrary or fitted parameters.

We find that for all columns, the first buckling mode to occur as strain is increased, i.e. the mode with smallest critical strain, is always either (i) a Euler mode, in which the column buckles in a direction perpendicular to the applied compression; or (ii) an alternating mode, in which adjacent holes are deformed into ellipses with orthogonal major axes, but the column remains straight overall.

The alternating mode is generally the first mode to become unstable under compression in columns of moderate length (≈4–16 holes) if the holes are relatively large, diameter greater than ≈75% of both the column width and hole spacing. For other column geometries, the Euler mode is the first mode to become unstable either via global buckling on the scale of the column (favoured in long columns or columns with small holes) or localized buckling on the scale of a hole (favoured when the ligaments adjoining the column edge are thinner than those separating holes). We have also studied the exchange of stability between the two modes by exploring the secondary bifurcations in the system.

Both Euler and alternating modes exhibit regimes of localized buckling, in which the buckling of the whole column occurs through buckling of thin ligaments around each hole, and at a critical strain that is nearly independent of the column length. This localized buckling of ligaments is captured by our asymptotic model, which provides an accurate quantitative description of the buckling when the holes are large. The model illustrates how the different types of buckling arise through deformation of the ligaments. The alternating mode involves bending of both those ligaments separating holes and those adjoining the column edges; this explains why the first buckling mode observed is the alternating mode only when both types of ligament are thin. By contrast, the Euler mode in its localized ‘sliding’ form involves buckling only of the ligaments adjoining the column edges, whereas in its global form the Euler mode involves the compression or extension, but not buckling, of these ligaments. The buckling behaviour of the column, and its dependence on the column geometry, can be understood and predicted from ligament deformations, as we demonstrate quantitatively for localized buckling in the alternating and Euler modes, equation (3.23). One reason for the success of the theory is that the buckling is strain-dominated and occurs at such small strains that material nonlinearities, and hence details of the constitutive law do not play a prominent role.

As shown in §4, these asymptotic ideas can be extended successfully to other elastic structures. Further extensions, for example, to more general grid structures [22], could allow this type of modelling to be used as a predictive tool for buckling in a wider range of perforated structures and metamaterials [4]. Such structures may be unstable to out-of-plane buckling modes [20], in addition to the in-plane modes we describe.

We find that, counterintuitively, the critical strain of Euler buckling in a column can be significantly increased by the presence of holes in a column. One avenue for future investigation is a more detailed study and experimental validation of this potentially useful phenomenon. Although the compressed column bifurcates first from the unbuckled compression branch only through the Euler or the alternating modes, we have shown that other bifurcations can occur at only slightly greater strains, notably higher-order Euler modes of buckling in the sliding regime. This invites the possibility of custom tuning of buckling states, through the addition of small geometrical changes or other imperfections in the system, or through dynamical effects. Most studies of buckling in cellular structures, to date, have considered only quasi-static compression of samples, but the dynamical compression is still largely unexplored, and could be of more practical relevance in large structures. A step in this direction was taken by Box *et al.* [23], who studied buckling in elastic and plastic cellular materials under dynamic compression, showing a considerable difference in the response of the latter depending on the rate of compression. This type of dynamic compression could, therefore, be explored with the aim of accessing modes other than the first Euler mode or the alternating mode in the holey column.

## Footnotes

1 Note that the critical strains for onset are sufficiently small that material nonlinearities have a minimal effect. The phenomenon appears to be entirely strain-driven and the use of different constitutive laws alters the critical stress, but the critical strains do not change significantly [12].

2 We note that a similar argument cannot be applied to the holey column, as the *w* ligaments have a straight edge on one side and a curved hole boundary on the other, and so are curved in their rest state. During the compression before the first bifurcation, these ligaments, therefore, bend outwards and compress, which complicates the evaluation of the effective Young’s modulus.

## Data accessibility

There are no experimental data associated with this work.
